# Photoinduced Electron Transfer from the Tryptophan Triplet State in Zn-Azurin

**DOI:** 10.1021/acsphyschemau.2c00042

**Published:** 2022-11-29

**Authors:** Joel J. Rivera, Christina Trinh, Judy E. Kim

**Affiliations:** Department of Chemistry and Biochemistry, University of California San Diego, La Jolla, California 92093, United States

**Keywords:** Tryptophan, azurin, electron transfer, proton transfer, phosphorescence, quantum yield, triplet, Stern−Volmer

## Abstract

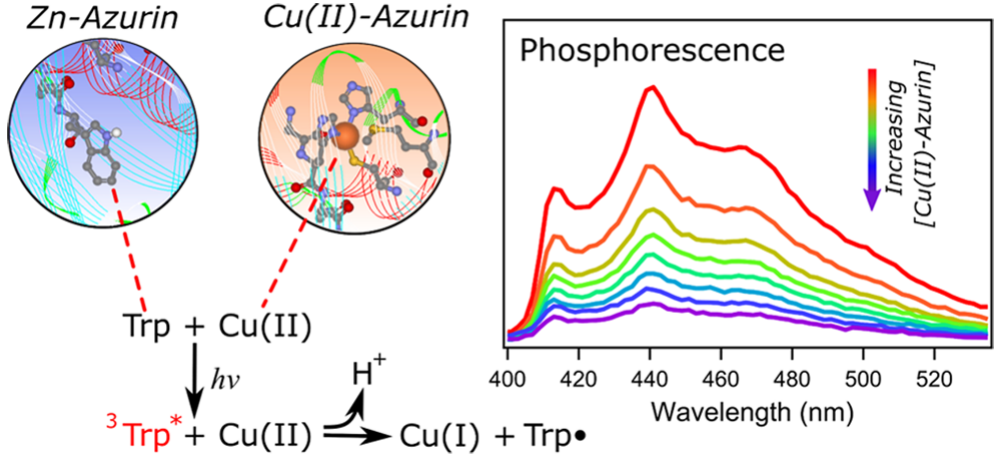

Tryptophan is one of few residues that participates in biological electron transfer reactions. Upon substitution of the native Cu^2+^ center with Zn^2+^ in the blue-copper protein azurin, a long-lived tryptophan neutral radical can be photogenerated. We report the following quantum yield values for Zn-substituted azurin in the presence of the electron acceptor Cu(II)-azurin: formation of the tryptophan neutral radical (Φ_rad_), electron transfer (Φ_ET_), fluorescence (Φ_fluo_), and phosphorescence (Φ_phos_), as well as the efficiency of proton transfer of the cation radical (Φ_PT_). Increasing the concentration of the electron acceptor increased Φ_rad_ and Φ_ET_ values and decreased Φ_phos_ without affecting Φ_fluo_. At all concentrations of the acceptor, the value of Φ_PT_ was nearly unity. These observations indicate that the phosphorescent triplet state is the parent state of electron transfer and that nearly all electron transfer events lead to proton loss. Similar results regarding the parent state were obtained with a different electron acceptor, [Co(NH_3_)_5_Cl]^2+^; however, Stern–Volmer graphs revealed that [Co(NH_3_)_5_Cl]^2+^ was a more effective phosphorescence quencher (*K*_SV_ = 230 000 M^–1^) compared to Cu(II)-azurin (*K*_SV_ = 88 000 M^–1^). Competition experiments in the presence of both [Co(NH_3_)_5_Cl]^2+^ and Cu(II)-azurin suggested that [Co(NH_3_)_5_Cl]^2+^ is the preferred electron acceptor. Implications of these results in terms of quenching mechanisms are discussed.

## Introduction

Long range electron transfer (ET) reactions can be enabled by redox-active intermediates that serve as electron “hopping spots” in biological systems.^1−3^ Organic and metal cofactors, such as flavin and heme, are examples of redox-active intermediates for long distance ET in biological systems.^4^ The aromatic amino acid residues tyrosine and tryptophan can also form radical intermediates in enzymes such as ribonucleotide reductase and DNA photolyase.^5,6^ Radical intermediates that involve proton transfer generally form through a concerted proton-coupled electron-transfer (PCET) reaction, where the proton and electron transfer reactions are considered simultaneous and a charged intermediate is not detectable, or a sequential mechanism, where the electron and proton are removed in discrete steps.^7^ In the latter case, a cation radical intermediate may or may not be detectable.

Several factors determine the mechanism of the PCET reaction. Tyrosine has a high p*K*_a_ and large, unfavorable oxidation potential, and this combination makes it challenging for tyrosine to exhibit a pure sequential mechanism in protein environments;^8,9^ however, experiments involving photosystem II have shown that a tyrosine neutral radical can form through a sequential mechanism.^10^ Regardless of the mechanism, the tyrosine cation radical is not observed in biological systems, largely because the cation radical has a very low p*K*_a_ that is not biologically accessible. A sequential PCET mechanism is thermodynamically accessible for tryptophan, and thus, tryptophan is typically oxidized to form the tryptophan cation radical^11,12^ in proteins or undergoes a two-step PCET reaction that yields a neutral radical.^13^ In model compounds, tryptophan radical intermediates can form through photoinduced PCET reactions where absorption of a photon generates an excited electronic state that serves as the parent state for ET and leads to the formation of a ground state cation radical intermediate;^14^ such mechanisms are different from photoinduced reactions where ET^15^ and PT^16,17^ may occur from the same excited electronic state.

The protein azurin is a model system for photoinduced PCET reactions involving tryptophan. Azurin is a small 14 kDa protein with a type 1 Cu(II) center and 128 residues that include a single tryptophan residue (W48) in a buried hydrophobic pocket. Previous studies have shown that photoexcitation of Zn-substituted azurin in the presence of an external electron acceptor generates a tryptophan neutral radical (W48•), which has been well characterized with experimental^18,19^ and computational techniques.^20^ The experimental studies showed that W48• is formed via an intermolecular reaction where the W48 residue in Zn-azurin reduces an extrinsic acceptor, which could be a Cu(II) metal center in a nearby Cu(II)-azurin or the Co(III) center of [Co(NH_3_)_5_Cl]^2+^. In the former case, the ligand-to-metal charge transfer (LMCT) band at 628 nm provides a spectroscopic signature that reflects the efficiency of the ET step separate from the proton transfer (PT) reaction.^21^ Furthermore, the long lifetime of W48• (7.3 h) and the characteristic absorption peak around 515 nm make it possible to quantify the radical quantum yield with steady state absorbance measurements.^19^

The mechanistic details of W48• formation in Zn-azurin are not fully understood but may involve photoinduced PCET between the triplet excited state, ^3^W48*, and an external electron acceptor. The idea of ET from ^3^W48* is consistent with prior studies. Long-lifetime, room temperature phosphorescence of azurin as well as other proteins has been reported.^22^ Several small molecules and proteins are known to quench tryptophan phosphorescence in azurin,^23−27^ and ET has been suggested as a phosphorescence quenching mechanism.^28,29^ A goal of this work is to demonstrate that ^3^W48* is the parent ET state for formation of W48• in Zn-azurin (we recently reported similar results for apoazurin).^30^ A Stern–Volmer analysis of phosphorescence quenching allows for the investigation of the quenching mechanisms with small molecule and protein quenchers; this analysis provides insights on protein–quencher interactions that affect the PCET reaction.

## Materials and Methods

### Sample Preparation

Buffers were prepared with purified water from a Barnstead NANOpure system (18.2 MΩ·cm). Phosphate buffer (KPi, 20 mM, pH 7.20) was prepared with K_2_HPO_4_ and KH_2_PO_4_ salts from Fisher Scientific. Sodium acetate buffer (50 mM, pH 4.50) was prepared with sodium acetate (99%) and acetic acid (99%) from Fisher Scientific. *N*-Acetyl-tryptophanamide (NATA, ≥98%) was purchased from Sigma-Aldrich. The electron acceptor [Co(NH_3_)_5_Cl]^2+^Cl_2_ (98%) was purchased from Sigma-Aldrich, and Cu(II)Cl_2_ (>99%) and Co(II)Cl_2_ (98.6%) were purchased from Fisher Scientific. ZnSO_4_ (99%) was purchased from EMD Millipore and CuSO_4_ (99%) was purchased from Alfa Aesar. All materials were used without further purification.

Azurin samples were expressed and purified as described in the literature.^31−33^ All azurin mutants in this study contained the single native tryptophan residue (W48) and no tyrosine residues (Y72F/Y108F). The metal center was removed using a potassium cyanide (≥98%, Fisher Scientific) dialysis procedure to generate the apoprotein.^21,33^ Apoprotein was remetalated via dialysis in 7 mM ZnSO_4_ or 7 mM CuSO_4_^21^ to generate Zn(II)-substituted azurin (ZnAzW48) or Cu(II)-azurin (CuAzW48). The reduced holoprotein with a Cu(I) center is referred to as Cu(I)AzW48.

The samples for emission and radical formation studies were comprised of 25 ± 2 μM ZnAzW48 and varying amounts of an electron acceptor (6–73 μM CuAzW48 or 3–60 μM [Co(NH_3_)_5_Cl]^2+^) in a total volume of 800 μL; the buffer composition was 90% KPi and 10% sodium acetate, with a final pH of 7.0. For experiments that involved the presence of both electron acceptors, the samples contained 25 ± 2 μM ZnAzW48, 31 ± 3 μM CuAzW48, and 4–34 μM [Co(NH_3_)_5_Cl]^2+^. Concentration-dependence emission experiments were performed on 10–56 μM ZnAzW48 in the absence of electron acceptor. Samples with CuCl_2_ acceptor contained 50 or 680 μM CuCl_2_ and 24 μM ZnAzW48 and were prepared in sodium acetate buffer. NATA samples with variable concentration were prepared in KPi.

### Steady-State Absorption and Emission Spectroscopy

If required, samples were deoxygenated prior to spectroscopic measurements. Oxygen was removed by evacuating the head space of the sample and backfilling with argon (99.999% AirGas) 25 times followed by gentle stirring of the sample under an argon atmosphere for 1 h. This pump-purge and stirring process was repeated three times, leading to a total deoxygenation process of approximately 3 h. The procedure was performed on a Schlenk line with an atmospheric- controlled 2 mm × 1 cm quartz cuvette. Absorption spectra were collected along the 1 cm path length using a Shimadzu UV-3600 spectrophotometer with a step size of 1.0 nm and bandpass of 1 nm. Emission spectra were collected with a JY Horiba Fluorolog-3 spectrofluorometer. The cuvette was oriented with the excitation beam passing through the 2 mm path and emission collected along the 1 cm path. The excitation and emission monochromators of the spectrofluorometer were set to a bandpass of 2 nm and a step size of 2.0 nm. The emission spectra were collected with 270 nm excitation to ensure that the highest energy features of the ZnAzW48 emission spectrum were recorded. Spectra of buffer-only were also acquired and subtracted from protein spectra to isolate emission from protein. Low temperature emission spectra were collected by cooling the cuvette carousel with a refrigerated water circulator and allowing the sample to equilibrate for 30 min before the spectrum was acquired. Direct measurement of the sample temperature was not possible because of the hermetic seals on the cuvette; however, parallel temperature measurements of cuvettes inserted into adjacent cells of the carousel revealed sample temperatures of 10 ± 1 °C (for low-temperature measurements) or 22 ± 1 °C (for room-temperature measurements).

### Generation of the Tryptophan Neutral Radical

The procedure for generation of W48• in ZnAzW48 with exogenous electron acceptor (CuAzW48 or [Co(NH_3_)_5_Cl]^2+^) has been described elsewhere.^19^ Briefly, a sample containing ZnAzW48 and the electron acceptor was deoxygenated before collecting the absorption and emission spectra that were used to calculate the emission quantum yield. The sample was photolyzed with dispersed light from a 450 W xenon short-arc lamp; the excitation wavelength was 292 nm with a monochromator bandpass of 10 nm and power at the sample of 0.13 mW. The sample was photolyzed for a total of ∼40 min while absorption spectra were collected at 1, 2, 3, or 5 min intervals. The concentration of W48• generated in these samples was 3–8 μM in the presence of CuAzW48 as the electron acceptor, 1–6 μM in the presence of [Co(NH_3_)_5_Cl]^2+^, and 2 μM in the presence of CuCl_2_. Experiments were performed to investigate the effect of UV power (0.05 to 0.84 mW) on the formation of the neutral radical; these power-dependence experiments revealed that 0.13 mW is within the range of optimal power (Supporting Information Figure S1).

### Calculation of Quantum Yields

Fluorescence and phosphorescence quantum yields were quantified using methods described in the literature^34^ with some adaptations to account for absorbance from nonemitting residues such as phenylalanine or the electron acceptor. Details of the calculations and relevant equations to generate normalized emission spectra are provided in the Supporting Information. Calculations for the radical quantum yield have been described previously.^19^ Similar methods were used to calculate Φ_rad_ and Φ_ET_ and are provided in the Supporting Information.

## Results

### Emission Spectra and Quantum Yields

**Figure 1:**
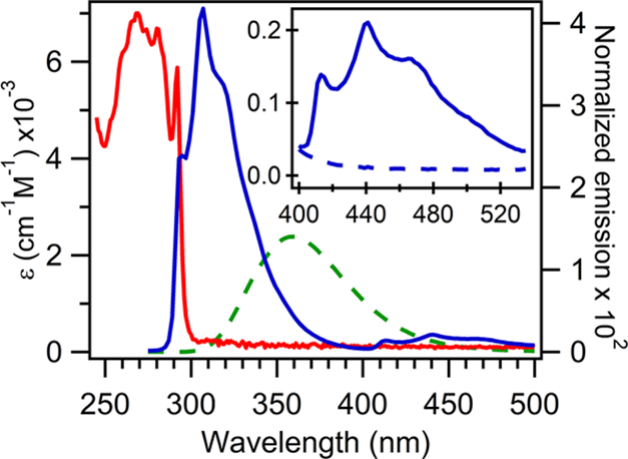
Absorption (red) and normalized emission (blue) spectra of deoxygenated ZnAzW48 in 20 mM KPi, pH = 7.0. The normalized emission spectrum of NATA in air is shown as the green dashed curve. The inset shows the phosphorescence region of deoxygenated ZnAzW48 (solid) and ZnAzW48 in air (dashed).

Absorption and normalized emission spectra of deoxygenated ZnAzW48 in 20 mM KPi, pH = 7.0, are shown in Figure 1; the normalized fluorescence spectrum of NATA in air is also shown for comparison. The values of the molar absorption coefficient were based on the published AzW48 value of 6690 M^–1^ cm^–1^ at 280 nm.^19^ The fluorescence and phosphorescence regions of ZnAzW48 appear at 275–400 nm and 400–535 nm, respectively. The relative intensities of the vibronic bands in the fluorescence^28,35^ and phosphorescence^24,28^ spectra agree with the literature; however the 293 nm vibronic band of the fluorescence spectrum is slightly attenuated by the overlapping 292 nm absorption peak (Figure S2). The subtle attenuation of the 293 nm band is negligible when calculating the total fluorescence quantum yield and thus, was not taken into account in the calculations. As described in the Supporting Information, the residual phosphorescence intensity above 535 nm was taken into account in calculations of the phosphorescence quantum yield. The fluorescence and phosphorescence quantum yields of ZnAzW48 were determined to be 0.21 ± 0.02 and 0.018 ± 0.001, respectively (Table 1). These values remained within 10% of each other over a concentration range of 10–56 μM (Figure S2).

**Table 1:** Quantum Yields for Fluorescence (Φ_fluo_), Phosphorescence (Φ_phos_), Neutral Radical Formation (Φ_rad_), and Electron Transfer (Φ_ET_) for ZnAzW48 in the Presence of CuAzW48 or [Co(NH_3_)_5_Cl]^2+^ Electron Acceptors^a^

[CuAz]/[ZnAz]	Φ_fluo_	Φ_phos_	Φ_rad_	Φ_ET_
Zn only^b^	0.21 ± 0.02	0.018 ± 0.001	−	−
0.45 ± 0.13^c^	0.19 ± 0.01	0.010 ± 0.002	0.17 ± 0.04	0.15 ± 0.04
0.98 ± 0.13^c^	0.19 ± 0.01	0.0063 ± 0.0008	0.22 ± 0.02	0.23 ± 0.01
1.4 ± 0.1^d^	0.19 ± 0.01	0.0050 ± 0.0005	0.25 ± 0.01	0.27 ± 0.01
2.4 ± 0.5^c^	0.19 ± 0.01	0.0030 ± 0.0005	0.30 ± 0.01	0.33 ± 0.02

[Co(III)]/[ZnAZ]	Φ_fluo_	Φ_phos_	Φ_rad_
0.11 ± 0.01^d^	0.21 ± 0.01	0.015 ± 0.001	0.15 ± 0.01
0.18 ± 0.01^d^	0.19 ± 0.01	0.011 ± 0.001	0.20^f^
0.25 ± 0.01^d^	0.19 ± 0.01	0.0094 ± 0.0006	0.20 ± 0.02
0.45 ± 0.03^e^	0.18 ± 0.01	0.0055 ± 0.0006	0.23 ± 0.01
2.0 ± 0.1^e^	0.19 ± 0.01	<0.0010	0.25 ± 0.01^g^

a At *T* = 22 °C. The concentration of ZnAzW48 was 25 ± 2 μM. Errors for *n* ≥ 3 are reported as one standard deviation. Entries with *n* = 2 are reported as the average with an error range that encompasses both values.

b *n* = 5.

c *n* = 4.

d *n* = 2.

e *n* = 3.

f *n* = 1.

g *n* = 7.

**Figure 2:**
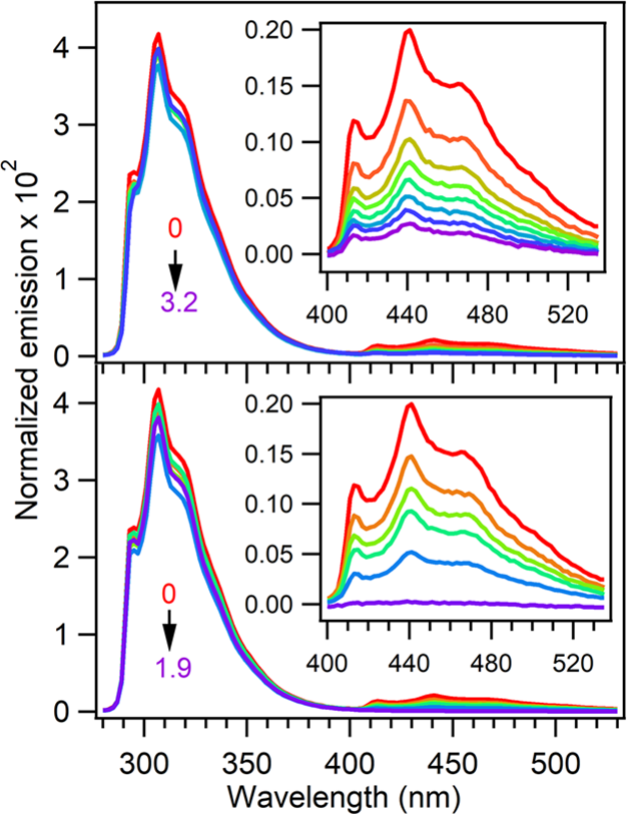
Normalized emission spectra of 25 ± 2 μM ZnAzW48 with increasing concentrations of electron acceptor CuAzW48 (top) and 26 ± 1 μM ZnAzW48 with increasing concentrations of [Co(NH_3_)_5_Cl]^2+^ (bottom). The ratios [CuAz]/[ZnAz] for these representative trials were 0, 0.26, 0.51, 0.80, 1.1, 1.5, 2.0, and 3.2, and [Co(III)]/[ZnAz] were 0, 0.10, 0.17, 0.24, 0.46, and 1.9. Insets show the expanded region of the phosphorescence.

Emission spectra of ZnAzW48 in the presence of increasing concentration of the electron acceptor were collected; similar ratios of ZnAzW48-to-acceptor were grouped, averaged, and summarized in Table 1. Figure 2 shows normalized fluorescence and phosphorescence spectra of a representative trial of 25 ± 2 μM ZnAzW48 with increasing concentration of CuAzW48 up to [CuAzW48]/[ZnAzW48] ≡ [CuAz]/[ZnAz] = 3.2. The tryptophan residue in CuAzW48 did not contribute significantly to Φ_fluo_ because CuAzW48 fluorescence is quenched by fast intramolecular ET from W48 to the Cu(II) metal center^35,36^ (Φ_fluo_ for CuAzW48 is 0.0043); a representative CuAzW48 fluorescence spectrum is shown in Figure S3. In contrast to the fluorescence signal, the phosphorescence of ZnAzW48 was quenched with increasing concentrations of CuAzW48. For example, in a representative trial of [CuAz]/[ZnAz] = 0.51, about 45% of the phosphorescence was quenched. At the highest ratio of [CuAz]/[ZnAz] = 3.2 of the representative trial, about 90% of the phosphorescence was quenched.

Analogous data were collected for ZnAzW48 with increasing concentration of [Co(NH_3_)_5_Cl]^2+^, and averaged values are included in Table 1. Representative emission spectra from a trial of 26 ± 1 μM ZnAzW48 with increasing concentration of [Co(NH_3_)_5_Cl]^2+^ up to ([Co(NH_3_)_5_Cl]^2+^/[ZnAzW48]) ≡ [Co(III)]/[ZnAz] = 1.9 are shown in Figure 2. The results are qualitatively similar to the CuAzW48 acceptor in that phosphorescence intensity decreased with increasing concentration of [Co(NH_3_)_5_Cl]^2+^ while fluorescence intensity was generally unaffected. However, [Co(NH_3_)_5_Cl]^2+^ was a more effective quencher than CuAzW48. For example, at a ratio of [Co(III)]/[ZnAz] = 0.46, more than 70% of the phosphorescence was quenched and at the highest ratio of 1.9, the phosphorescence was fully quenched.

**Figure 3:**
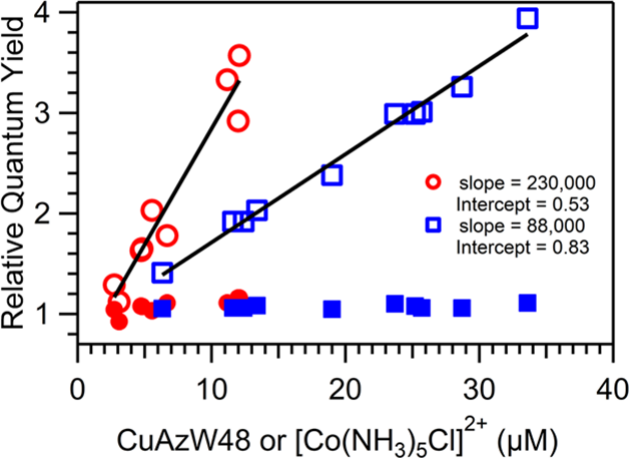
Stern–Volmer plot of ZnAzW48 showing the relative phosphorescence (Φ_phos_^0^**/**Φ_phos_, open markers) and relative fluorescence (Φ_fluo_^0^**/**Φ_fluo_, filled markers) quantum yields with acceptors CuAzW48 (blue squares) and [Co(NH_3_)_5_Cl]^2+^ (red circles) at *T* = 22 °C. The phosphorescence data were fit to the linear Stern–Volmer equation (solid line), and the resulting values of *K*_SV_ (slope, units M^–1^) and *y*-intercepts are indicated. Data are shown up to [CuAz]/[ZnAz] =1.5 and [Co(III)]/[ZnAz] = 0.46.

The individual values of Φ_fluo_ and Φ_phos_ that led to the average values in Table 1 were used to generate Stern–Volmer plots for fluorescence and phosphorescence of ZnAzW48 with CuAzW48 and [Co(NH_3_)_5_Cl]^2+^ electron acceptors in Figure 3. The value of Φ_fluo_^0^/Φ_fluo_, where Φ_fluo_^0^ is the fluorescence in the absence of acceptor, was constant at approximately 1 for all concentrations of acceptor. In contrast, the value of Φ_phos_^0^/Φ_phos_ increased linearly with increasing acceptor concentration. A linear fit to the graphs of Φ_phos_^0^/Φ_phos_ vs acceptor concentration revealed Stern–Volmer quenching constants (*K*_SV_) of 88 000 M^–1^ and 230 000 M^–1^ for the acceptors CuAzW48 and [Co(NH_3_)_5_Cl]^2+^, respectively. These slopes reflect data up to [CuAz]/[ZnAz] = 1.5 and [Co]/[ZnAz] = 0.46 where the signal was reliable. Intercepts from the linear fits were 0.83 for CuAzW48 and 0.53 for [Co(NH_3_)_5_Cl]^2+^; these intercept values deviate from the expected value of 1.0 and could, in part, reflect the limited range of quencher concentrations, particularly for the [Co(NH_3_)_5_Cl]^2+^ data which is collected over a small range of concentration. The presence of additional quenching mechanisms could also impact the intercept (see below). The values of Φ_fluo_ and Φ_phos_ were also determined at 10 °C (Supporting Information Table S1); the value Φ_phos_^0^/Φ_phos_ for a sample with [Co(III)]/[ZnAz] = 0.26 increased from 1.8 at 22 °C to 7.2 at 10 °C, however the values of Φ_phos_^0^/Φ_phos_ for [CuAz]/[ZnAz] = 0.57 (*T* = 10 °C) or 0.51 (*T* = 22 °C) are nearly identical at 2.0 and 1.9, respectively. The value of Φ_fluo_^0^/Φ_fluo_ remained approximately 1 at both temperatures for both acceptors.

### Radical Formation and Quantum Yields

**Figure 4:**
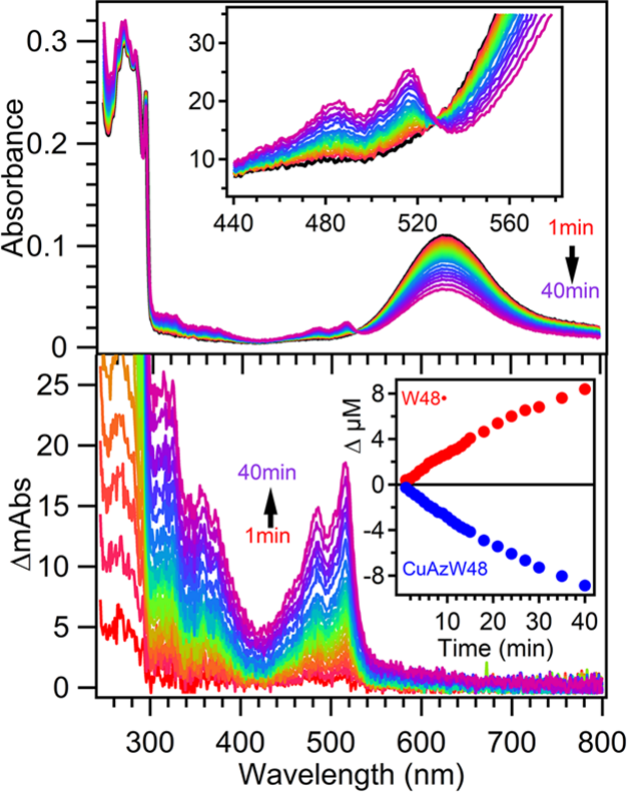
Top panel: Representative absorption spectra during the photolysis of ZnAzW48 in the presence of CuAzW48 with [CuAz]/[ZnAz] = 0.80 for 1 to 40 min. The black curve is the prephotolysis spectrum. Inset shows the region of the isosbestic point. Bottom panel: Difference spectra calculated by subtracting the prephotolysis spectrum from each spectrum on the top panel. The difference spectra have been corrected for the 628 nm bleach. The inset shows the increase in concentration of W48• in ZnAzW48 and decrease in concentration (bleach) of the Cu(II) metal center in CuAzW48.

Representative spectra collected during photolysis of a protein sample with [CuAz]/[ZnAz] = 0.80 are shown in Figure 4. The 488 and 515 nm absorption bands have been assigned to W48• that is produced in ZnAzW48 through a PCET reaction with CuAzW48 as the electron acceptor.^18,19^ Photolysis of CuAzW48 alone does not produce neutral radical (Figure S4). In Figure 4, the growth in the region 300–400 nm is assigned to W48• because the rate of growth is identical to the main peak near 514 nm (Figure S5). Decay of the 628 nm absorbance band reflects reduction of Cu(II) to Cu(I) in CuAzW48; the slight loss of Cu(II) signal with direct photoexcitation of CuAzW48 in the absence of ZnAzW48 was not taken into account because of the small magnitude of this bleach (Figure S4). Neither Cu(I)AzW48 nor W48• absorb at 628 nm, and thus, the decay at this wavelength provides a direct measurement of CuAzW48 reduction, leading to Φ_ET_.

**Figure 5:**
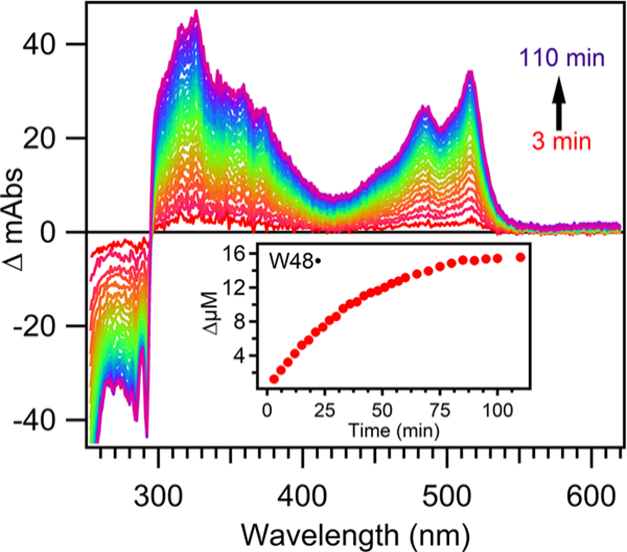
Representative difference spectra for the photolysis of ZnAzW48 in the presence of [Co(NH_3_)_5_Cl]^2+^ with [Co(III)]/[ZnAz] = 1.9 for 3 to 110 min. The inset shows the increase in concentration of W48•.

Representative difference spectra during photolysis of ZnAzW48 with [Co(III)]/[ZnAz] = 1.9 are shown in Figure 5. The growth of W48• is indicated by the 515 and 488 nm peaks. The decay at wavelengths shorter than 300 nm reflects depletion of W48 upon conversion to W48• combined with loss of the 228 nm absorption band of [Co(NH_3_)_5_Cl]^2+^. Because of the overlap in absorbance by ZnAzW48, [Co(NH_3_)_5_Cl]^2+^, and possibly W48• in this UV region, the loss of signal from [Co(NH_3_)_5_Cl]^2+^ could not be used to determine Φ_ET_ with [Co(NH_3_)_5_Cl]^2+^ as the acceptor.

The values of Φ_rad_ and Φ_ET_ with varying amounts of CuAzW48 or [Co(NH_3_)_5_Cl]^2+^ are presented in Table 1. The ratio, Φ_rad_/Φ_ET_, reflects the probability to generate the neutral radical following an ET event; this quantity is referred to as the efficiency of PT of the cation radical (Φ_PT_). The values for Φ_PT_ are high, greater than 0.91 for all values of [CuAz]/[ZnAz], with an average value of 0.98 ± 0.10. These high values for Φ_PT_ indicate that nearly every ET event is followed by a PT event to produce the neutral radical W48•. The values for Φ_rad_ with [Co(NH_3_)_5_Cl]^2+^ as acceptor also increased with higher values of [Co(III)]/[ZnAz], with a max value of Φ_rad_ = 0.25.

**Figure 6:**
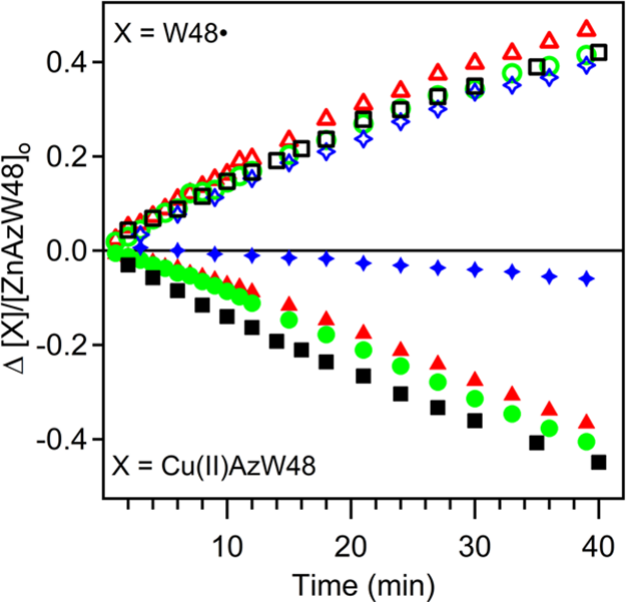
Growth of the 515 nm absorbance (top, open symbols) and decay of the 628 nm absorbance (bottom, filled symbols) during photolysis of ZnAzW48 in the presence of CuAzW48 or both [Co(NH_3_)_5_Cl]^2+^ and CuAzW48 electron acceptors. The concentrations are as follows: Black squares [ZnAzW48] = 23 μM and [CuAzW48] = 29 μM; green circles [ZnAzW48] = 24 μM, [CuAzW48] = 29 μM, and [Co(NH_3_)_5_Cl]^2+^ = 4 μM; red triangles [ZnAzW48] = 26 μM, [CuAzW48] = 31 μM, and [Co(NH_3_)_5_Cl]^2+^ = 7 μM; blue stars [ZnAzW48] = 28 μM, [CuAzW48] = 35 μM, and [Co(NH_3_)_5_Cl]^2+^ = 34 μM.

Figure 6 shows results from the photolysis of ZnAzW48 in the presence of both [Co(NH_3_)_5_Cl]^2+^ and CuAzW48 acceptors; the corresponding Φ_rad_ and Φ_ET_ values were calculated from linear fits to the first 15 or 16 min of photolysis (Table S2). The values of [CuAz]/[ZnAz] were 1.2–1.3, and increasing amounts of [Co(NH_3_)_5_Cl]^2+^ were added to achieve [Co(III)]/[ZnAz] = 0.17, 0.27, or 1.2. The value of Φ_rad_ remained unchanged at 0.27 ± 0.02 upon addition of [Co(NH_3_)_5_Cl]^2+^; however, the value of Φ_ET_ decreased significantly and systematically from 0.26 to 0.035. These competition experiments indicate that in the presence of equimolar [Co(NH_3_)_5_Cl]^2+^ and CuAzW48, nearly all ET events proceeded with [Co(NH_3_)_5_Cl]^2+^ as the acceptor, not CuAzW48. In the presence of 5× excess CuAzW48 compared to [Co(NH_3_)_5_Cl]^2+^ (where [Co(III)]/[ZnAz] = 0.27 and [CuAz]/[ZnAz] = 1.2), about half the ET events proceeded through CuAzW48 because the value of Φ_PT_ was 2.0 (from 0.28/0.14); a value of Φ_PT_ greater than 1.0 is obviously not possible and indicates that at least half the PT events are not accounted for by CuAzW48 alone. With even a lower concentration of [Co(NH_3_)_5_Cl]^2+^ such that there is 8× excess CuAzW48 compared to [Co(NH_3_)_5_Cl]^2+^ ([Co(III)]/[ZnAz] = 0.17 and [CuAz]/[ZnAz] = 1.2), a greater fraction of the ET events proceeded through CuAzW48, evidenced by the decrease in Φ_PT_ to 1.4 (from 0.27/0.20), a value closer to the theoretical maximum of 1.0. For this experiment, in the limit of no [Co(NH_3_)_5_Cl]^2+^ and only CuAzW48, the value of Φ_PT_ is 0.96, which is within the error of the average value described above.

## Discussion

The ability to generate a stable tryptophan neutral radical in azurin can be compared to model compounds. For tryptophan in water, the yield of electron ejection with 280 nm excitation is 0.07 and increases to 0.22 with ∼220 nm excitation into the B_b_ band; this photoejection has been proposed to occur from a singlet excited state.^37^ In azurin, the yields of electron ejection and formation of the neutral radical of W48 with 292 nm excitation are as high as 0.3, suggesting that the protein enables efficient ET to generate the stable neutral radical.

The emission properties of azurin have been studied extensively. The fluorescence spectrum has an emission maximum at 307 nm, which is known to be the most blue-shifted of all proteins; a typical solvent-exposed tryptophan residue emits around 350 nm.^38^ The phosphorescence properties of azurin are also unique compared to other proteins. In deoxygenated solutions, W48 in wild-type azurin exhibits room-temperature phosphorescence with a long lifetime of 400 ms,^22^ which contrasts with a phosphorescence lifetime of ∼40 μs to ∼1 ms for the model compound tryptophan in water.^39,40^ The blue-shifted fluorescence and long phosphorescence lifetime have been attributed to the rigid and solvent-excluded, nonpolar environment around W48.^38,41^ This unique environment is likely responsible for the unusual stability of W48• because the absence of a nearby proton source prevents efficient back-ET to regenerate the closed-shell, protonated W48 species.

The temperature-dependence of phosphorescence and fluorescence quantum yields is known. In the present study, the ratio of phosphorescence intensity at 465 nm (*I*_phos_^465^) to fluorescence intensity at 320 nm (*I*_fluo_^320^) at room temperature for ZnAzW48 is 0.050. Previous reports suggested that the lifetime-corrected phosphorescence quantum yield, Φ_phos_(*T*)/τ_phos_(*T*), is independent of temperature^28,42^ and that the ratio of the intersystem quantum yield (Φ_ISC_) to fluorescence quantum yield (Φ_fluo_) is constant from low temperature (150 K or −123 °C) to room temperature.^42^ To the best of our knowledge, there are no reports of the absolute phosphorescence quantum yields of azurin. However, one of these previous studies indicated a low-temperature ratio of phosphorescence to fluorescence for apoazurin (wild type) as 0.087.^28^ Taking into account the ∼14-fold difference in triplet lifetimes at low- (140 K or −133 °C) vs room-temperature of 5.6 and 0.41 s, respectively, the present ratio of phosphorescence to fluorescence of 0.050 is an order of magnitude larger than the previous result of 0.006 when the previous result is extrapolated to room temperature (from 0.087/5.6 s = 0.0155 and 0.0155 × 0.41 s = 0.006). This difference is not attributed to the metal centers (Zn-protein in the current study vs apoprotein in the earlier report) because the phosphorescence and fluorescence yields of ZnAzW48 and apoAzW48 are similar (Table S3). The reason for the discrepancy in ratio of quantum yields between the present and prior experiments is not clear and may reflect experimental variations (e.g., deoxygenation method).

The quenching properties have also been investigated. It is well-known that the fluorescence of W48 is quenched in the native holoazurin because of intramolecular ET from W48* to the Cu(II) center.^35,36^ Phosphorescence quenching of wild-type apoazurin and various metalated systems, for example, Cd- and Zn-substituted wild-type azurin (CdAzWT and ZnAzWT, respectively) has also been reported using extrinsic molecular quenchers such as acrylamide,^23−25^ O_2_,^25^ H_2_S and HS^–^,^26^ as well as protein–protein interactions between Cu(I)AzWT and the oxidized Cu(II) form of CuAzWT.^28^

### Phosphorescence Quenching

The Stern–Volmer graphs indicate that the phosphorescence of ZnAzW48, and not fluorescence, is quenched in the presence of CuAzW48 and [Co(NH_3_)_5_Cl]^2+^ electron acceptors. The efficiency of phosphorescence quenching (Φ_phos_^0^/Φ_phos_) is linear as a function of acceptor concentration, and thus, the quenching constant (*K*_SV_) can be interpreted as an association constant (*K*_a_) for a static quenching mechanism or bimolecular quenching term (*k*_q_*τ*_0_) for a dynamic quenching mechanism (*k*_q_ is the bimolecular quenching constant and τ_0_ is the lifetime of the fluorophore in the absence of quencher).

Previous reports of azurin phosphorescence lifetimes have indicated a purely dynamic (collisional) mechanism for quenching. These prior studies focused on wild-type azurin with different metal centers, for example, CdAzWT, ZnAzWT, and apoAzWT; these variants exhibited intense phosphorescence relative to the native CuAzWT. Measurements of triplet lifetimes with small-molecule, neutral phosphorescence quenchers, such as acrylamide and O_2_, indicate a linear decrease in lifetime with increasing concentration of quencher.^25^ This relationship supports a dynamic quenching mechanism. Phosphorescence lifetime quenching of CdAzWT in the presence of CuAzWT was also linear, indicating that the quenching mechanism with a protein quencher is also dynamic.

Results from the previous study of CdAzWT + CuAzWT can be compared with the present experiments of ZnAzW48 + CuAzW48. The bimolecular quenching constant, *k*_q_, for a mixture of CdAzWT donor and CuAzWT quencher was reported as 5.3 × 10^4^ M^–1^ s^–1^, corresponding to a value of *K*_SV_ of 2.7 × 10^4^ M^–1^ (using a phosphorescence lifetime τ_0_ of 0.51 s for CdAzWT).^28^ The analogous *K*_SV_ and *k*_q_ values for ZnAzW48 in the presence of CuAzW48 are 8.8 × 10^4^ M^–1^ and 1.6 × 10^5^ M^–1^ s^–1^, respectively, assuming a phosphorescence lifetime of 0.55 s^23^ for ZnAzW48 based on reported lifetime for ZnAzWT. These *k*_q_ values of 10^4^ to 10^5^ M^–1^ s^–1^ for azurin are consistent with typical protein–protein association kinetics of 10^4^ to 10^6^ M^–1^ s^–1^ based on diffusional association.^43^ The ability of the copper center in CuAzW48 to quench the phosphorescence of W48 in ZnAzW48 while being reduced to Cu(I) indicates that CuAzW48 is within interaction distance of W48 in ZnAzW48 within the lifetime of the triplet state and that the association persists long enough for ET to take place; implications of these rate constants in terms of protein binding kinetics are discussed below.

The mechanism of quenching ZnAzW48 phosphorescence with the cationic charged electron acceptor, [Co(NH_3_)_5_Cl]^2+^, is less clear. All the prior studies focused on neutral or anionic quenchers. A comparison of the lifetime of the azurin triplet with H_2_S and HS^–^ indicated a 100× decrease in the value of *k*_q_, from 1.8 × 10^4^ M^–1^ s^–1^ for H_2_S to less than 1 × 10^2^ M^–1^ s^–1^ for HS^–^.^26^ The significant decrease in the value of *k*_q_ for the anionic quencher was attributed to the inability of the anionic quencher to diffuse to W48.^26^ As the size of a neutral quencher increased, the value of *k*_q_ decreased from 3.1 × 10^3^ (acrylonitrile) to 1.5, 2.7, and 0.5 M^–1^ s^–1^ (acrylamide and its derivatives).^26^ These values can be compared to quenching with O_2_, which has the largest *k*_q_ value of 1.3 × 10^7^ M^–1^ s^–1^. For reference, the freely diffusing model compound tryptophan with acrylamide exhibits a *k*_q_ value of 5.9 × 10^9^ M^–1^ s^–1^.^44^ The decrease in *k*_q_ for azurin compared to the model compound is attributed to the need for the quencher to diffuse to W48. Given that [Co(NH_3_)_5_Cl]^2+^ is charged and much larger than acrylamide, one might expect a relatively low quenching efficiency, that is, a small value for the Stern–Volmer constant, compared to acrylamide. The opposite is observed: *K*_SV_ for [Co(NH_3_)_5_Cl]^2+^ is 2.3 × 10^5^ M^–1^. If *K*_SV_ were interpreted strictly in terms of a dynamic mechanism, the value of *k*_q_ would be 4.2 × 10^5^ M^–1^ s^–1^ (assuming a phosphorescence lifetime of 0.55 s), which is larger by a factor of 10^5^ than that of acrylamide and also more than 20-fold larger than that of a small molecule, H_2_S. These results suggest that the quenching of ZnAzW48 phosphorescence with [Co(NH_3_)_5_Cl]^2+^ may not involve a purely dynamic quenching mechanism.

The exceptionally large value of *K*_SV_ for [Co(NH_3_)_5_Cl]^2+^ could reflect dominance of a static mechanism. In this case, *K*_SV_ would be interpreted as an association constant, *K*_a_, instead of a purely bimolecular quenching constant. Temperature dependence measurements of Φ_phos_^0^/Φ_phos_ are consistent with a static quenching mechanism. The efficiency to quench ZnAzW48 phosphorescence with [Co(NH_3_)_5_Cl]^2+^ increased from 1.8 at room temperature to 7.2 at 10 °C; this 4-fold increase in Φ_phos_^0^/Φ_phos_ primarily reflects a substantial drop in Φ_phos_ by a factor of 0.36 at 10 °C compared to room temperature while Φ_phos_^0^ only slightly increased by a factor of 1.4 upon reduction of temperature (Supporting Information Table S1). These temperature findings are consistent with static quenching where the bound complex is stabilized at the lower temperature.^44^ A purely dynamic mechanism would have the opposite effect where a lower temperature results in decreased quenching. Specifically, the decrease in temperature and accompanying increase in viscosity^45^ would lead to a drop in the calculated diffusion coefficient of a factor of about 1.4 based on the Stokes–Einstein equation. In comparison, there was no detectable temperature dependence for CuAzW48 over the same temperature range; Φ_phos_^0^/Φ_phos_ with CuAzW48 were 1.9 and 2.0 at 22 °C and 10 °C, respectively. This lack of an observable temperature dependence with CuAzW48 may reflect the combined effects of temperature on diffusion constant and yields of ET and PT. This type of interpretation where the reaction is considered purely dynamic or purely static is simplistic because there is both a diffusion-controlled component and equilibrium consideration in the quenching process; for electron self-exchange reactions of azurin, the diffusion controlled, bimolecular exchange rate (*k*_ex_) has been described in terms of the equilibrium constant and inherent ET rate (*k*_ex_ = *K*_a_*k*_ET_).^46^ Thus, an increase in *K*_a_ at a lower temperature for [Co(NH_3_)_5_Cl]^2+^ would lead to the observed increase in the diffusion-controlled value for *k*_ex_.

The [Co(NH_3_)_5_Cl]^2+^ electron acceptor was more efficient than CuAzW48. A solution with [Co(III)]/[ZnAz]) = 0.25 quenched approximately half of the phosphorescence while a ratio of 0.46 quenched 70%. This level of efficiency was not observed with CuAzW48, where a representative trial of [CuAz]/[ZnAz] = 0.51 quenched about half the phosphorescence. We were unable to identify alternative quenching mechanisms attributed to [Co(NH_3_)_5_Cl]^2+^. The reduction of [Co(NH_3_)_5_Cl]^2+^ is an irreversible process that leads to the formation of [Co(H_2_O)_6_]^2+^ in solution.^47^ We explored the possibility that the Co(II) product or dissociated amine ligands could quench the triplet via study of a mixture of CoCl_2_ + ZnAzW48 as well as NH_4_Cl + ZnAzW48; neither of these mixtures quenched the phosphorescence of W48 (Figure S6). Another possibility is the heavy metal effect, which is known to enhance intersystem crossing; however, this quenching mechanism typically requires close interaction between the triplet molecule and the external heavy atom.^48^ W48 resides in a solvent-excluded, hydrophobic cavity that is 8 Å from the aqueous phase^49^ and is unlikely to interact closely with [Co(NH_3_)_5_Cl]^2+^. In the case of metal-substituted azurins, such as Co(II), Hg(II), Cd(II), and Ag(I), phosphorescence lifetimes remain similar to those of apoazurin and ZnAzWT despite a distance of around 10 Å between the metal and W48.^23,27,28^ Structural changes near W48 are unlikely because there are no modifications in the shape or position of the fluorescence and phosphorescence spectra in the presence of [Co(NH_3_)_5_Cl]^2+^. We compared the effect of an alternate external quencher, CuCl_2_, and found that CuCl_2_ quenched the phosphorescence more efficiently than CuAzW48, but not as efficiently as [Co(NH_3_)_5_Cl]^2+^ (Table S4).

The origin of the strong quenching ability of [Co(NH_3_)_5_Cl]^2+^ (∼2-fold more efficient than CuAzW48) remains unknown. There may be a component in the mechanism that allows [Co(NH_3_)_5_Cl]^2+^ to interact with multiple ZnAzW48 and quench ^3^W48* efficiently, perhaps through the formation of a dark complex that could also affect the intercept of the Stern–Volmer graph. Strong binding between the Co(III) complex and ZnAzW48 is not unreasonable given that azurin is expected to be overall negatively charged at pH = 7 (pI value of 5.6).^50^ The different paths and distances for the two quenchers are expected to play an important role in the ET process despite similar reduction potentials for [Co(NH_3_)_5_Cl]^2+^ (anodic peak of 0.3 V vs NHE^51^) and CuAzW48 (0.31 V vs NHE^52^). We continue to investigate the origin of the strong quenching with lifetime studies.

### Protein Binding Kinetics

As mentioned above, the characterization of the quenching mechanism in the presence of CuAzW48 as purely collisional is too simplistic for azurin because the quenching process of ET requires a minimum interaction time during which a transient dimer is formed and ET takes place. An additional complication is that in a mixture of ZnAzW48 and CuAzW48, three transient dimers may be formed: a heterodimer of ZnAzW48+CuAzW48 (ZnCu) and two homodimers (CuCu and ZnZn). Only the heterodimer is active in terms of quenching; CuCu exhibits no phosphorescence and the ZnZn homodimer does not quench phosphorescence in the present range of concentrations (Figure S2). In this analysis, we assume that there is no thermodynamic preference for the heterodimer vs homodimers. While direct kinetic measurements of interprotein ET are not available for the ZnCu dimer, we estimate the ET rate constant as 10^6^ s^–1^ based on the equation *k*_max_ = 10^13^ s^–1^ exp{−β(*R* – 3 Å)} where β is the distance-decay constant (β = 1.1 Å^–1^) and R is the donor–acceptor separation estimated to be 16–18 Å.^53^ This ET rate is much shorter than the lifetime of the triplet but may compete with the dimer dissociation rate. Ultimately, ET will only take place if every triplet is able to sample the heterodimer within the lifetime of the triplet state.

The idea of a transient azurin dimer has been explored in previous studies of the interprotein electron self-exchange (e.s.e.) reaction between Cu(I) and Cu(II) sites in a mixture of reduced and oxidized wild-type azurin. These studies suggested that the protein units interact via the hydrophobic patch in azurin and that the self-exchange rate is about 1 × 10^6^ M^–1^ s^–1^ at room temperature.^54−56^ In these Cu(I)AzWT–Cu(II)AzWT transient dimers, the presence of interfacial water and conformational flexibility are believed to play an important role in the efficiency of the ET reaction over the >15 Å distance between the two copper atoms.^57−59^ An NMR study of the e.s.e. reaction suggested a value of 1 M^–1^ for the association constant.^46^ This low value of the association constant is consistent with the encounter complex in 1–2 mM azurin as assessed by NMR as well as the primarily monomeric population of azurin up to 130 μM based on phosphorescence anisotropy experiments.^46,60^

The kinetic on- and off-rates for the dimer can be estimated. The Stern–Volmer graph indicates a bimolecular rate constant, *k*_q_, of 1.6 × 10^5^ M^–1^ s^–1^; this value can be interpreted as *k*_on_ in terms of the association constant for the heterodimer, *K*_ZnCu_. Assuming a value of 1 M^–1^ for *K*_ZnCu_ based on prior studies of e.s.e. reactions, the value of 1/*k*_off_ would be 1.6 × 10^5^ s^–1^. The lifetime of the dimer is 1/*k*_off_, which is 6 μs. This lifetime is comparable to the ET rate and short enough to enable sampling of the homodimers and heterodimer in the relatively long ∼0.5 s lifetime of the triplet state. Based on these relevant rates, it is feasible for the ZnAzW48 + CuAzW48 mixture to exhibit efficient quenching of the triplet state via ET.

The situation with the mixture of ZnAzW48 + [Co(NH_3_)_5_Cl]^2+^ is more complicated. Nonetheless, a similar analysis can be performed to obtain limiting values of the lifetime of the azurin-Co(III) complex. In this case, the value of *K*_SV_ can be interpreted as an association constant for the complex, *K*_a_, and is 2.3 × 10^5^ M^–1^. The large value for *K*_a_ suggests the lifetime of the complex is relatively long. If the bimolecular constant takes on a value of an upper limit of 10^9^ M^–1^ s^–1^, the value of *K*_off_ becomes 4300 s^–1^, leading to a lower limit of the lifetime (shortest lifetime) of the complex of 230 μs; this lifetime is longer than the ET rate. Lower values of the bimolecular constant would lead to lifetimes of the complex longer than 230 μs; for example, a more realistic bimolecular constant of 10^6^ M^–1^ s^–1^ leads to a lifetime of the complex of 230 ms.

### Φ_rad_ and Φ_ET_

**Figure 7:**
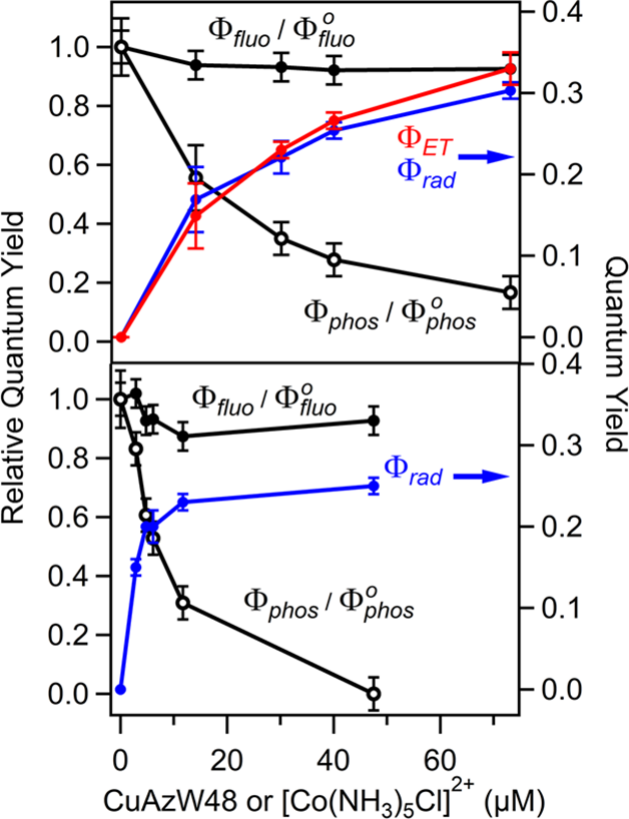
Left axis: Relative quantum yields for fluorescence (Φ_fluo_/Φ_fluo_^0^, black filled circle) and phosphorescence (Φ_phos_/Φ_phos_^0^, black open circle) for ZnAzW48 in the presence of CuAzW48 (top panel) and [Co(NH_3_)_5_Cl]^2+^ (bottom panel). Right axis (blue and red curves): Quantum yields for radical formation (Φ_rad_, blue filled circle) and ET (Φ_ET_, red filled circle). Lines connect the points to help guide the eye.

The values of Φ_rad_ are shown in Figure 7. For the CuAzW48 electron acceptor, the values of Φ_ET_ are also shown. The quantity Φ_PT_ = Φ_rad_/Φ_ET_ is greater than 0.91 for all concentrations of CuAzW48, indicating that essentially every ET event is followed by a PT event to generate the neutral radical. There is an observed anticorrelation between Φ_ET_ and Φ_phos_ in which Φ_ET_ increases as Φ_phos_ decreases (the same anticorrelation exists between Φ_rad_ and Φ_phos_); in the same range of CuAzW48 concentrations, Φ_fluo_ remains relatively constant. While the analogous values of Φ_ET_ and Φ_PT_ could not be obtained with [Co(NH_3_)_5_Cl]^2+^, a similar anticorrelation between Φ_rad_ and Φ_phos_ as well as a constant value for Φ_fluo_ were also observed. These observations for Φ_ET_, Φ_rad_, Φ_phos_, and Φ_fluo_ as a function of concentration of electron acceptor suggest that the phosphorescent state is the precursor to W48• for both electron acceptors.

Additional evidence for the triplet state of W48 as the parent state for ET is provided by the isotope effects measured with ZnAzW48-*d*_5_, which contains the perdeuterated W48 isotopologue with five nonexchangeable deuterium atoms on the indole ring. Emission spectra of ZnAzW48-*d*_5_ with 291.5 nm excitation have previously been reported,^21^ and in the present study, the fluorescence spectra were measured with 270 nm excitation in order to compare the results of ZnAzW48-*d*_5_ with those of protiated ZnAzW48. In the absence of quencher, the isotope effects Φ_fluo_^D^/Φ_fluo_^H^ and Φ_phos_^D^/Φ_phos_^H^ were 0.95 and 0.72, respectively, indicating that perdeuteration reduced Φ_phos_ without affecting fluorescence (Figure S7). In the presence of similar ratios of [CuAz]/[ZnAz], the isotope effects for fluorescence and phosphorescence were 1.0 and 0.80, respectively; the isotope effects for radical formation (Φ_rad_^D^/Φ_rad_^H^) and ET (Φ_ET_^D^/Φ_ET_^H^) were nearly identical to each other, 0.83 and 0.82, respectively. The similarity of the radical and ET yields indicates that perdeuteration does not affect the PT yield. More importantly, the similarity of the isotope effects for Φ_phos_, Φ_rad_, and Φ_ET_ combined with a negligible isotope effect for Φ_fluo_ provide additional evidence that in the presence of CuAzW48 as the electron acceptor, ET takes place from the phosphorescent triplet state. A similar result was found with [Co(NH_3_)_5_Cl]^2+^ as the electron acceptor (Figure S7).

Competition experiments in which ZnAzW48 was photolyzed in the presence of both [Co(NH_3_)_5_Cl]^2+^ and CuAzW48 revealed a preference for [Co(NH_3_)_5_Cl]^2+^ as the electron acceptor. In these experiments, the ratio [CuAz]/[ZnAz] was held constant and increasing amounts of [Co(NH_3_)_5_Cl]^2+^ were added. The data indicate that the formation of W48• is unaffected by the addition of [Co(NH_3_)_5_Cl]^2+^, but the ET process to CuAzW48 is nearly eliminated with equimolar amounts of [Co(NH_3_)_5_Cl]^2+^ and CuAzW48. In this situation where both electron acceptors are present [Co(NH_3_)_5_Cl]^2+^ could interact with CuAzW48. However, this interaction does not appear to affect the formation of the neutral radical. Despite the preference for [Co(NH_3_)_5_Cl]^2+^ over CuAzW48 for ET, the value of Φ_rad_ in the presence of only [Co(NH_3_)_5_Cl]^2+^ is lower than that of CuAzW48. This finding motivated our search for an alternative quenching mechanism that does not involve ET to [Co(NH_3_)_5_Cl]^2+^, but we were unable to identify such a mechanism (described above).

Differences in the rate of electron transfer (*k*_ET_) and the rate of proton transfer (*k*_PT_) may also contribute to lower Φ_rad_ values with [Co(NH_3_)_5_Cl]^2+^. For example, the *k*_ET_ values for [Co(NH_3_)_5_Cl]^2+^ and CuAzW48 will be affected by different ET paths and distances on account of variation in binding interactions. Differences in *k*_PT_ could also contribute to smaller Φ_rad_ values for [Co(NH_3_)_5_Cl]^2+^. PT pathways have been proposed for the formation of W48• in ZnAzW48,^18^ but it is unknown if the formation of a CuAzW48–ZnAzW48 dimer facilitates or obstructs this pathway. It is interesting that both Φ_rad_ and Φ_phos_ are lower with CuCl_2_ as an electron acceptor relative to CuAzW48 (Table S4). This observation suggests that CuCl_2_ is capable of more efficient quenching of the phosphorescence from ZnAzW48 than CuAzW48, potentially because of enhanced ET, but the PT step may be hindered as evidenced by the lower Φ_rad_ compared to CuAzW48. One possible reason for the decreased PT efficiency with CuCl_2_ is the absence of a protein–protein dimer that may be needed to form the neutral radical. A similar effect could contribute to lower Φ_rad_ values for [Co(NH_3_)_5_Cl]^2+^ relative to CuAzW48.

### Implications for the Quantum Yield for Intersystem Crossing

**Figure 8:**
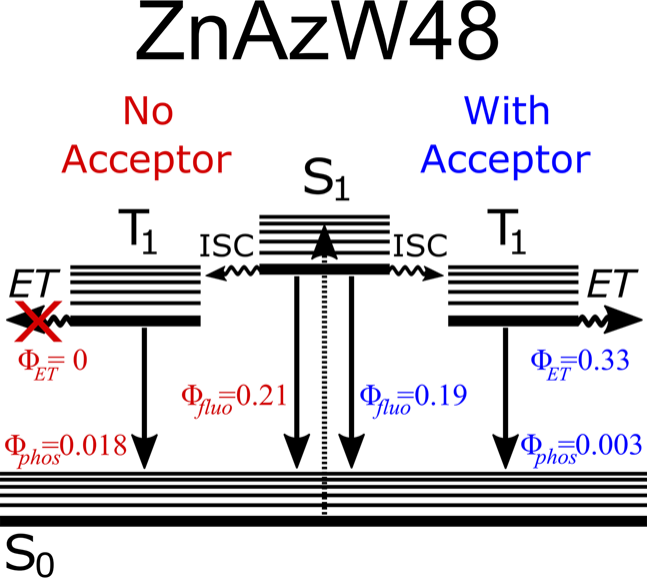
Relevant relaxation pathways and measured quantum yields for photoexcited W48 in ZnAzW48 in the presence (right path) and absence (left path) of electron acceptor; all values are from Table 1. In the presence of the CuAzW48 electron acceptor [CuAz]/[ZnAz] = 2.4 ± 0.5), the triplet state relaxes via ET to the Cu(II) metal center and the value of Φ_phos_ is negligible at 0.003. In the absence of an electron acceptor, ET from T_1_ is not possible and Φ_phos_ is high at 0.018. In contrast to Φ_phos_, the value of Φ_fluo_ is not significantly affected by the presence of an electron acceptor.

The result for Φ_ET_ provides a lower limit for the quantum yield for intersystem crossing (Φ_isc_). In our analysis, measurements of Φ_ET_ are based on the number of ET events per photon absorbed by W48 in ZnAzW48. The radiative and nonradiative relaxation pathways are summarized in Figure 8. Assuming that all ET events originate from the triplet state, the largest value of Φ_ET_ is a lower limit for Φ_isc_. Our results indicate that the value of Φ_isc_ is at least 0.33. The efficiency of intersystem crossing is expected to be identical in the presence of either electron acceptor. The value of 0.33 for Φ_isc_ can be compared to prior measurements of model compounds. Studies of indole reported Φ_isc_ values of 0.23 in water and 0.43 in cyclohexane at room temperature;^61^ in a supersonic free jet, the value was 0.3.^62^ The values for Φ_isc_ for aqueous l-Trp or NATA are generally lower than indole but also variable, reported from 0.065 to 0.27.^63−65^ Despite the uncertainty in literature values, the Φ_isc_ value of 0.33 reported here is not unreasonable, especially given that the value may be higher in a hydrophobic pocket as indicated from the comparison of indole in water and cyclohexane.

## Conclusion

The photogeneration of W48• in ZnAzW48 was investigated in the presence of two different electron acceptors. Comparison of the Φ_ET_ and Φ_rad_ values to Φ_phos_ and Φ_fluo_ indicates that the parent state of ET is the triplet, not singlet, state of W48. CuAzW48 quenches the phosphorescence in a dynamic manner, and this quenching is concomitant with formation of W48• in ZnAzW48 as well as reduction to Cu(I)AzW48. [Co(NH_3_)_5_Cl]^2+^ also quenches the phosphorescence of ZnAzW48, and is about 2-fold more efficient than CuAzW48; despite this enhanced efficiency, Φ_rad_ is lower with [Co(NH_3_)_5_Cl]^2+^ than with CuAzW48. These studies highlight the critical role of the triplet state in ET reactions that involve tryptophan.

## Supporting Information Available

Determination of quantum yields and generation of normalized fluorescence spectra; power dependence graph for the radical quantum yield; concentration-dependence of the emission quantum yields emission and photolysis of CuAzW48; comparison of the growth of 358 and 515 nm of the radical; emission spectra in the presence of Co(II)Cl_2_ or NH_4_Cl; data for ZnAzW48-*d*_5_; tables of various quantum yields (PDF)
