# Supplementary material for: Photoinduced Electron Transfer from the Tryptophan Triplet State in Zn-Azurin
Source: ACS Phys Chem Au. 2022 Nov 29;3(1):63–73. doi: 10.1021/acsphyschemau.2c00042 (PMC9881450; doi:10.1021/acsphyschemau.2c00042)
Supplement: Supplementary file 1 — pg2c00042_si_001.pdf [file pg2c00042_si_001.pdf]

Supporting Information

Photoinduced electron transfer from the tryptophan triplet state in Zn-azurin

*Joel J. Rivera, Christina Trinh, and Judy E. Kim**

Department of Chemistry and Biochemistry, University of California San Diego, La Jolla, CA

Email: judyk@ucsd.edu

Determination of quantum yields. NATA was used as the reference to determine the fluorescence and phosphorescence quantum yields for ZnAzW48. The excitation wavelength and bandpass were identical for NATA and ZnAzW48 spectra. The concentration of NATA was optimized such that the NATA absorbance was similar (within 3%) to the protein sample at the excitation wavelength. This optimization ensured that the protein and NATA samples experienced similar primary inner filter effects and the spectra could be analyzed with minimal additional corrections. Re-absorption of emitted light (secondary inner filter effect) was not taken into account because the sample absorbance at wavelengths above 310 nm is negligible.

The literature value of 0.13 for the fluorescence quantum yield of NATA[1] was used to calculate the fluorescence quantum yield (Φ_{fluo}) and phosphorescence quantum yield (Φ_{phos}) of ZnAzW48. The analysis for determination of emission quantum yields was adapted from the literature[2] and modified to account for the presence of absorbing, but not emitting, moieties such as phenylalanine or the electron acceptor. The raw fluorescence spectrum was initially corrected by subtraction of the buffer-only spectrum from the raw spectrum; the integrated intensity of this corrected fluorescence spectrum for W48 is denoted $\int_{275}^{400} Fluo_{corr} d\lambda$ and can be expressed as:

$$\int_{275}^{400} Fluo_{corr}\, d\lambda = \left(I_0\left[1 - 10^{-(Abs_x \times \ell_x)}\right]\right) \times \left(\frac{Abs_{W48}}{Abs_{solution}}\right) \times \Phi_{fluo} \qquad (1)$$

A similar expression can be written for NATA:

$$\int_{300}^{500} Fluo_{NATA,corr} d\lambda = \left(I_0\left[1 - 10^{-(Abs_x \times \ell_x)}\right]\right) \times \Phi_{fluo,NATA} \qquad (2)$$

In both Eqs. (1) and (2), the first term on the right, $I_0\left[1 - 10^{-(Abs_x \times \ell_x)}\right]$, reports the light absorbed by the solution where I_0 is the intensity of incident light and the term $1 - 10^{-(Abs_x \times \ell_x)}$ is the fraction of excitation light absorbed through the pathlength of the sample cuvette; this is the estimated sampling position of the collection optics. Abs_x is the absorbance of sample x (where x is NATA or the protein solution) at the excitation wavelength of 270 nm over a 1-cm pathlength and ℓ_x is the pathlength for the excitation beam through the cuvette ($\ell_{NATA} = \ell_{protein} = 0.2\ cm$).

For experiments with ZnAzW48 and CuAzW48, there is a second term that reports the fraction of absorbed light attributed to the photoactive/emitting chromophore of interest, which is W48 in ZnAzW48, relative to all absorbing species, including non-emitting W48 in CuAzW48 and phenylalanine residues; this second term is the ratio of absorbances of W48 in ZnAzW48 to

the solution. This ratio is $\frac{Abs_{W48}}{Abs_{solution}} = \frac{\varepsilon_{W48}[ZnAzW48](\ell=1)}{\varepsilon_{protein}[protein](\ell=1)}$ where ε_{W48} and $\varepsilon_{protein}$ are the molar attenuation coefficients for W48 and the protein sample, respectively. The quantity $[ZnAzW48]$ is identical to the concentration of W48 in ZnAzW48, and $[protein]$ is the concentration of all proteins in the solution. In the case of ZnAzW48 in the absence of quencher, $[ZnAzW48] = [protein]$ and this ratio $\frac{Abs_{W48}}{Abs_{protein}}$ becomes $\frac{\varepsilon_{W48}}{\varepsilon_{protein}}$. If the sample contains both ZnAzW48 and the electron acceptor CuAzW48, the denominator $[protein] = [ZnAzW48] + [CuAzW48]$. The relevant values of molar extinction coefficients are provided below.

Collectively, the product of the first two terms on the right side of Eq. (1) is referred to as the light absorbed by W48 and the first term on the right side of Eq. (2) is referred to as the light absorbed by NATA. The final spectra presented here have been normalized for the amount of light absorbed by the chromophore of interest as well as emission from NATA on the same day. The reason for inclusion of NATA emission on the same day is because all measurements of the fluorescence quantum yield are referenced to NATA, and day-to-day variations must be taken into account. See below for further details on the normalization process. The value of I_0 is identical in both Eqs. 1 and 2, and the value of Φ_{NATA} is 0.13.[1] The final equation for Φ_{fluo} for W48 in ZnAzW48 is:

$$\Phi_{fluo} = \frac{\int_{275}^{400} Fluo_{corr} d\lambda}{\left[1-10^{-\left(Abs_{protein}\times 0.2\right)}\right]\times\frac{Abs_{W48}}{Abs_{solution}}} \times \frac{\left[1-10^{-\left(Abs_{NATA}\times 0.2\right)}\right]}{\int_{300}^{500} Fluo_{NATA,corr} d\lambda} \times 0.13 \qquad (3)$$

The integral in the numerator of Eq. 3 was slightly modified for the phosphorescence quantum yield for W48 in ZnAzW48 (Φ_{phos}). The phosphorescence spectrum contains residual fluorescence that elevates the baseline of the phosphorescence spectrum, especially near 400 nm. This residual fluorescence was removed by subtracting the emission spectrum of the sample in the presence of oxygen, where phosphorescence is fully quenched, from the emission spectrum of the deoxygenated sample where fluorescence is unaffected by oxygen. This difference spectrum was integrated from 400-535 nm to determine the integrated area of ZnAzW48 phosphorescence, i.e., the integral term in Eq. 1 became $\int_{400}^{535} Phos_{diff} d\lambda$ where $Phos_{diff}$ represents the phosphorescence difference spectrum. An additional correction factor of 1.04 was multiplied by the integrated area to account for residual phosphorescence at wavelengths to the red of 535 nm. Emission spectra could not be recorded at wavelengths longer than 535 nm

because second order light from the 270 nm excitation light appeared at 540 nm. The correction factor of 1.04 was determined from emission spectra collected with 291.5 nm excitation where the second order excitation light does not affect the phosphorescence spectrum. Thus, the expression for the phosphorescence quantum yield is:

$$\Phi_{phos} = \frac{1.04 \times \int_{400}^{535} Phos_{diff}}{\left[1-10^{-\left(Abs_{protein} \times 0.2\right)}\right] \times \frac{Abs_{W48}}{Abs_{solution}}} \times \frac{\left[1-10^{-\left(Abs_{NATA} \times 0.2\right)}\right]}{\int_{300}^{500} Fluo_{NATA,corr}} \times 0.13 \qquad (4)$$

The radical quantum yield (Φ_{rad}) and electron transfer quantum yield (Φ_{ET}) values were determined from absorption spectra of mixtures of ZnAzW48 and an electron acceptor. First, difference spectra were generated by subtracting the pre-photolysis spectrum (absorption spectrum of the sample before photolysis) from each post-photolysis spectrum at various stages of photolysis. The disappearance of the 628 nm band on account of the reduction of CuAzW48 to Cu(I)AzW48 produced a negative bleach feature in the difference spectra. The bleach was used to determine the loss of CuAzW48 using a value of $\varepsilon_{\mathrm{CuAzW48}}$ of 5900 M^{-1} cm^{-1} at 628 nm.[3] The loss of CuAzW48 on account of reduction of Cu(II) is equivalent to gain of Cu(I)AzW48 and a linear fit of the formation of Cu(I)AzW48 in the first 15 or 16 minutes of photolysis is the numerator in the equation $\Phi_{ET} = \frac{rate\ of\ CuAzW48\ reduction}{rate\ of\ excitation\ of\ W48}$. The denominator is the rate of excitation of W48 in ZnAzW48, and this term has been described previously.[4] To determine Φ_{rad}, the absorption feature at 515 nm from ZnAzW48• had to be isolated from the bleach at 628 nm because these regions partially overlapped. The overlap of the 628 nm and 515 nm features was corrected by adding a fraction of the pre-photolysis spectrum to the difference spectrum at a given timepoint such that the bleach of the 628 nm band was eliminated and the 650 to 800 nm region was flat. Because each difference spectrum exhibited a different amount of bleach, variable fractions of the pre-photolysis spectrum were needed to eliminate the bleach; these spectra in which the bleach was eliminated are referred to as corrected difference spectra (see below for an example). The intensities of the 515 nm peaks in the corrected difference spectra were plotted against total photolysis time to generate a single wavelength kinetic trace. The literature value of 2200 M^{-1} cm^{-1} at 515 nm for $\varepsilon_{\mathrm{ZnAzW48\bullet}}$ was used to convert the absorbance to concentration of ZnAzW48•.[4] A linear fit of the initial region (up to 16 min) of the increase in [ZnAzW48•] was used to calculate the rate of formation of ZnAzW48• in the equation $\Phi_{rad} = \frac{rate\ of\ formation\ of\ \mathrm{ZnAzW48\bullet}}{rate\ of\ excitation\ of\ W48}$. The denominator is the same value used for Φ_{ET}.

In experiments with $[Co(NH_3)_5Cl]^{2+}$ or $CuCl_2$ as the acceptor, there was no bleach at 628 nm and thus, corrected difference spectra did not need to be generated.

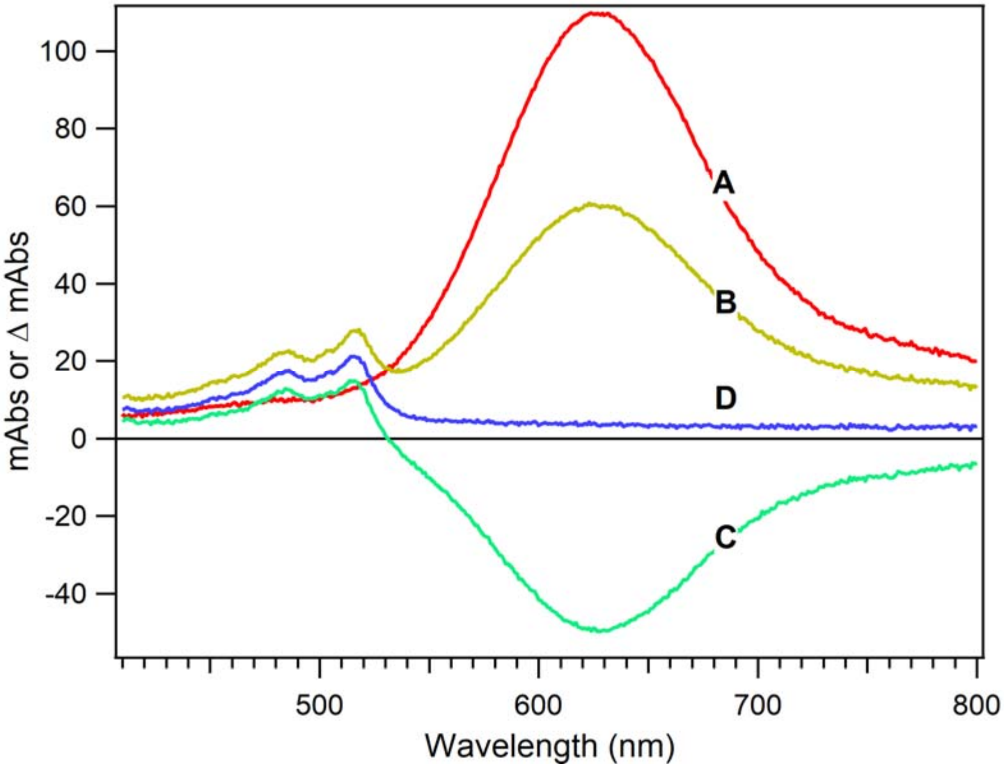

Above: Absorption and difference absorption spectra of a deoxygenated mixture of CuAzW48 and ZnAzW48 at a ratio $[CuAz]/[ZnAz] = 0.80$. Trace A is measured before photolysis and Trace B is collected 40 min after photolysis with 292 nm. Trace C is the difference spectrum calculated from $C = B - A$, the positive features at 515 and 488 nm are attributed to growth of the neutral radical while the negative feature centered at 628 nm shows the bleach of the Cu(II) absorbance band after reduction to Cu(I). Trace D is the corrected difference spectra calculated from $D = C + \alpha A$ where α is the scalar value required to remove the negative feature at 628 nm. Different values of α are required for different time points.

Process to generate normalized fluorescence spectra. The corrected fluorescence spectrum of NATA is normalized for amount of absorbed light via the wavelength-dependent

function $\frac{Fluo_{NATA,corr}(\lambda)}{\left[1-10^{-(Abs_{NATA}\times 0.2)}\right]}$, and the normalized integrated intensity for NATA is $\frac{\int_{300}^{500} Fluo_{NATA,corr} d\lambda}{\left[1-10^{-(Abs_{NATA}\times 0.2)}\right]}$. The final normalized spectrum of NATA shown in Figure 1 is

$$\frac{Fluo_{NATA,corr}(\lambda)}{\left[1-10^{-(Abs_{NATA}\times 0.2)}\right]} \times \frac{\left[1-10^{-(Abs_{NATA}\times 0.2)}\right]}{\int_{300}^{500} Fluo_{NATA,corr} d\lambda} = \frac{Fluo_{NATA,corr}(\lambda)}{\int_{300}^{500} Fluo_{NATA,corr} d\lambda}$$

and the area under the NATA curve in Figure 1 is equal to 1.0.

The normalized emission spectra of ZnAzW48 in the main text were analyzed in an analogous manner by normalizing the corrected fluorescence spectrum of W48 to the amount of light absorbed by W48 as well as the emission of NATA on the same day. Thus, the final spectra referred to as normalized emission for W48 are given by the wavelength-dependent function:

$$\frac{Fluo_{corr}(\lambda)}{\left[1-10^{-(Abs_{protein}\times 0.2)}\right] \times \frac{Abs_{W48}}{Abs_{solution}}} \times \frac{\left[1-10^{-(Abs_{NATA}\times 0.2)}\right]}{\int_{300}^{500} Fluo_{NATA,corr} d\lambda}$$

The ratio of integrated areas for W48 and NATA is equal to the ratio of fluorescence quantum yields of these two species.

Relevant molar extinction coefficients for Φ_{phos} and Φ_{fluo} calculations. The values of molar extinction coefficients at 270 nm used in Equations 1-4 are $\varepsilon_{W48} = 5900$ cm^{-1} M^{-1} and $\varepsilon_{protein} = 7060$ cm^{-1} M^{-1} if the protein sample contains only ZnAzW48 or $\varepsilon_{protein} = 7060 + 7060 = 14{,}120$ cm^{-1} M^{-1} if the sample contains both ZnAzW48 and CuAzW48. We note that the value for ε_{W48} reflects only the tryptophan residue; this value was determined by subtracting the molar extinction coefficient of the all-phe mutant W48F/Y108F/Y72F of 1160 cm^{-1} M^{-1} from that of ZnAzW48 (i.e., $\varepsilon_{W48} = 7060 - 1160 = 5900$ cm^{-1} M^{-1}). In experiments where $[Co(NH_3)_5Cl]^{2+}$ is the quencher, the denominator $Abs_{solution}$ is the absorbance of the sample of ZnAzW48 + $[Co(NH_3)_5Cl]^{2+}$; the contribution of $[Co(NH_3)_5Cl]^{2+}$ to the absorbance at 270 nm is included ($\varepsilon_{\mathrm{Co(NH_3)_5Cl}} = 830$).

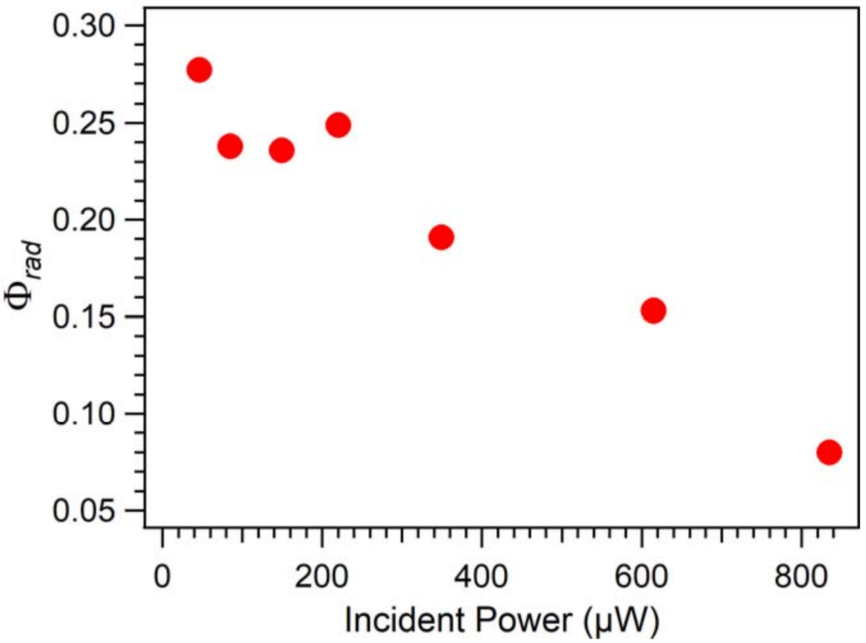

Figure S1. Power dependence curve for Φ_{rad} of ZnAzW48 with the electron acceptor $[Co(NH_3)_5Cl]^{2+}$. The average ratio $[Co(III)]/[ZnAz]$ for all trials is 2.00 ± 0.04.

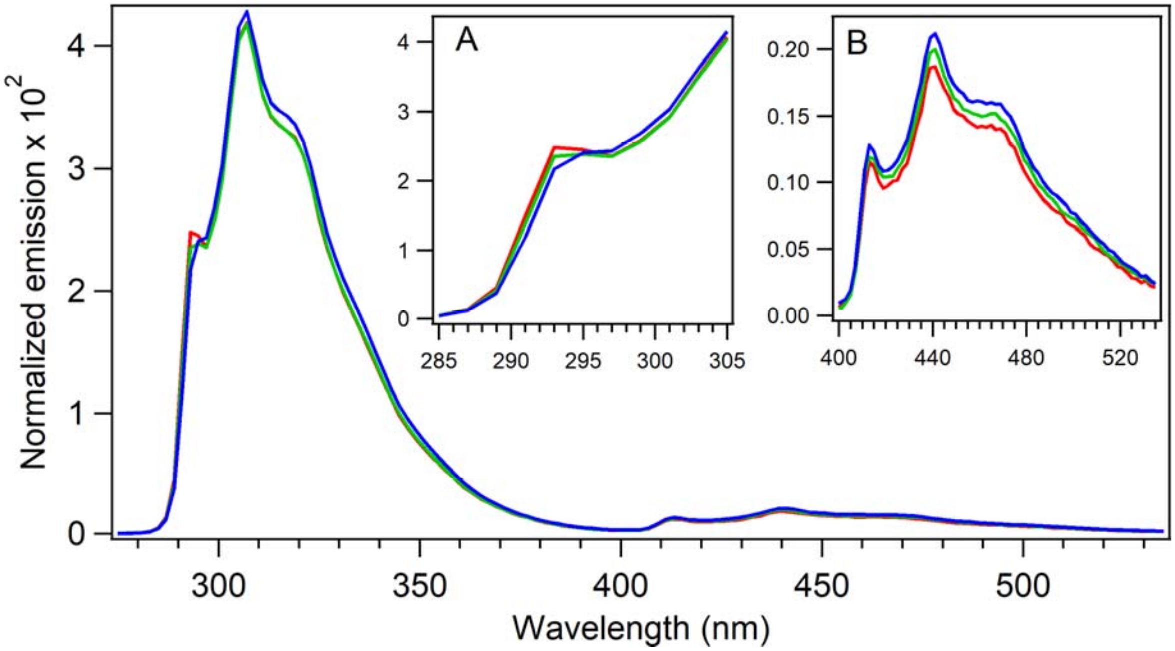

[ZnAzW48] (µM)	Φ_{fluo}	Φ_{phos}
10	0.21	0.017
27	0.21	0.019
56	0.22	0.020

Figure S2. Representative spectra of ZnAzW48 samples with [ZnAzW48] =10 µM (red), 27 µM (green), and 56 µM (blue) in the absence of electron acceptors. All spectra have been normalized as described in the text. The fluorescence and phosphorescence spectra are nearly identical with the exception of the 293 nm peak that is attenuated at higher concentrations as shown in inset A. The phosphorescence region is shown as inset B. The table shows Φ_{fluo} and Φ_{phos} values at these different concentrations of ZnAzW48 in the absence of acceptor.

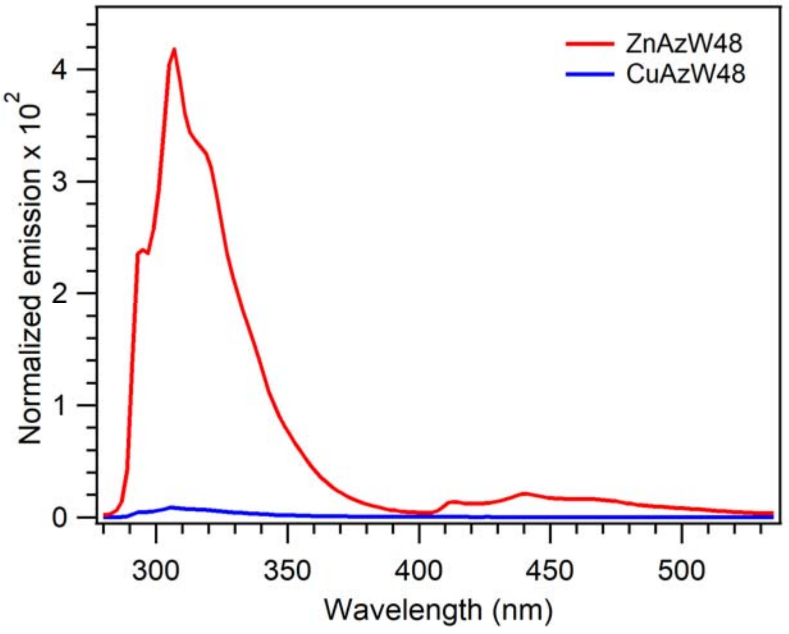

Figure S3. Emission spectra of deoxygenated pure ZnAzW48 and pure CuAzW48 with 270 nm excitation. Spectra are normalized as described in the text. The Φ_{fluo} values are 0.21 and 0.0043 for ZnAzW48 and CuAzW48 respectively. There are no distinguishable features in the phosphorescence region (400-535 nm) for CuAzW48.

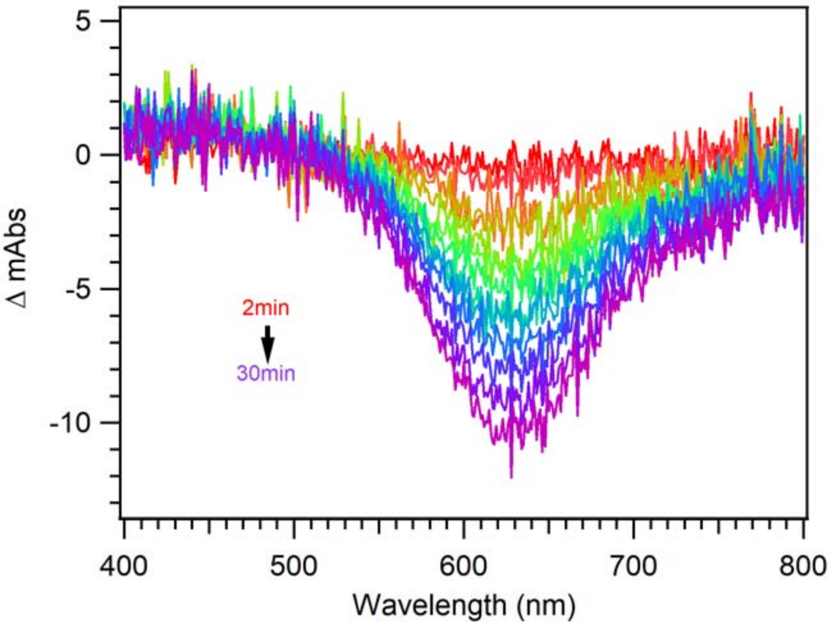

Figure S4. Difference spectra from the photolysis of deoxygenated CuAzW48 (33 μM) for 2-30 minutes. The characteristic W48• peaks at 514 and 488 nm are not observed in these spectra. The negative feature centered at 628 nm indicates photodecay of the CuAzW48 protein. The quantum yield of CuAzW48 photodecay can be estimated as $\Phi_{decay} = \frac{rate\ of\ CuAzW48\ reduction}{rate\ of\ excitation\ of\ W48}$ and has a value of 0.033 ± 0.001 ($n = 3$).

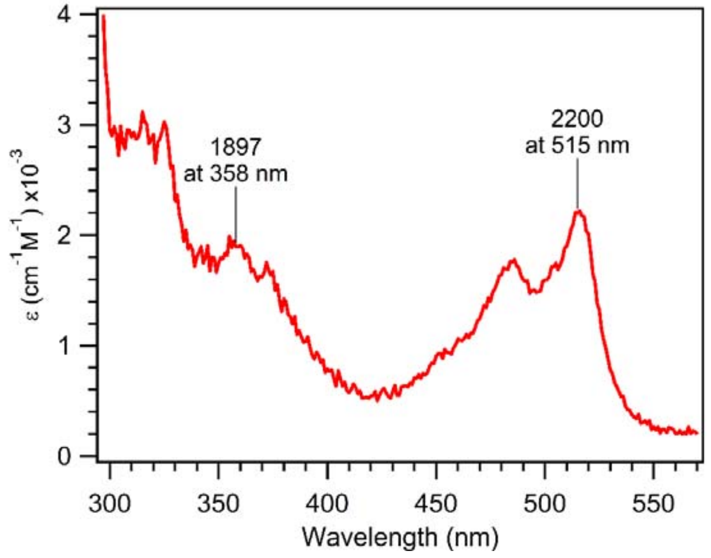

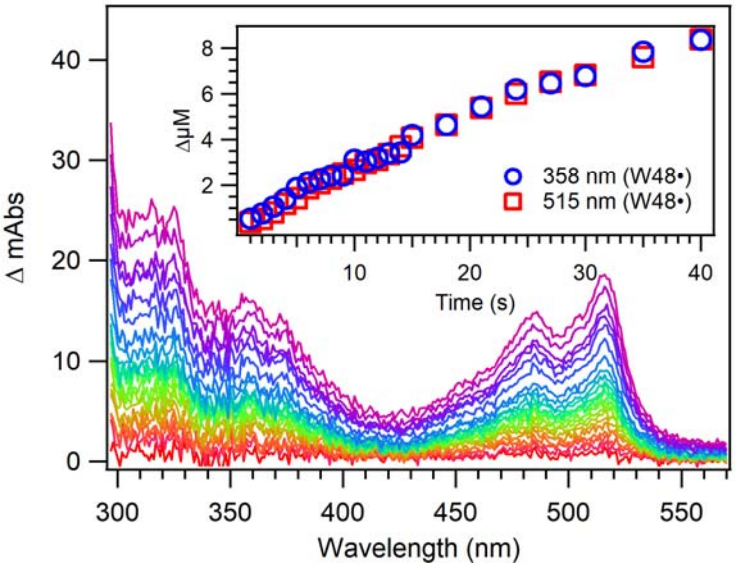

Figure S5. Top: Corrected difference spectrum from a sample of ZnAzW48 with $[CuAz]/[ZnAz] = 0.80$ after 40 minutes of photolysis. The value of the molar absorption coefficient at 358 nm was determined from the literature value of 2200 cm^{-1} M^{-1} at 515 nm for W48•. Bottom: Corrected difference spectra from the photolysis of ZnAzW48 with $[CuAz]/[ZnAz] = 0.80$. The inset shows the growth of W48• based on the intensity of the 515 and 358 nm features.

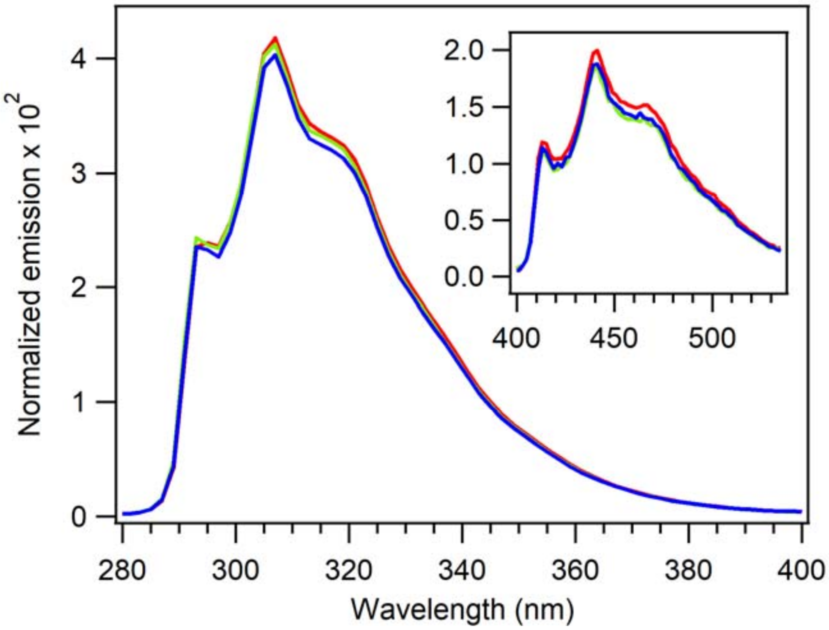

Figure S6. Normalized fluorescence spectra of deoxygenated ZnAzW48 with $[Co(II)Cl_2]/[ZnAz] = 3.2$ (green), $[NH_4Cl]/[ZnAz] = 8.0$ (blue), and ZnAzW48 only (red); the data are from a single trial. The Φ_{fluo} values are 0.21 with Co(II)Cl_2 , 0.20 with NH_4Cl, and 0.21 for ZnAzW48 only. The phosphorescence spectra are not affected by the quenchers as shown in the inset. The Φ_{phos} values are 0.017, 0.017 and 0.018 for samples with Co(II)Cl_2, NH_4Cl, and ZnAzW48 only, respectively.

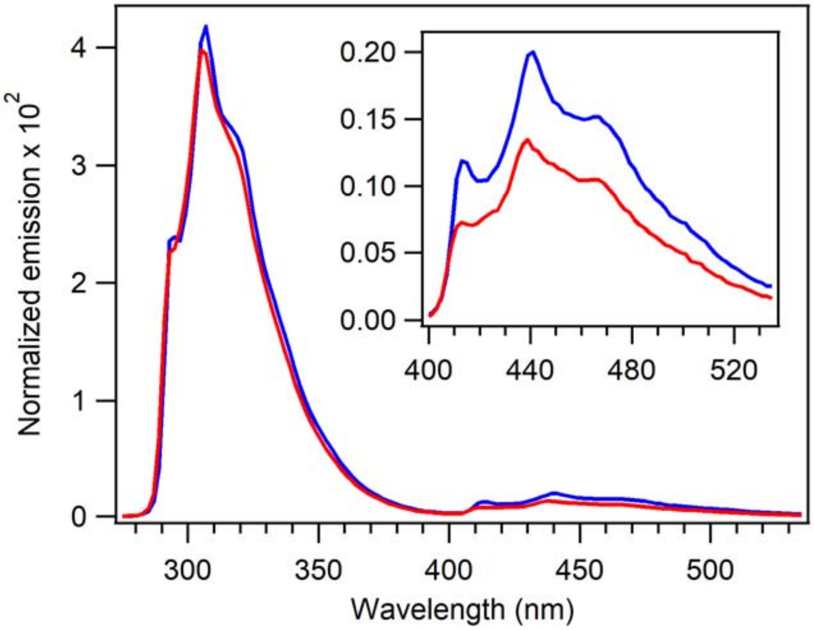

Acceptor	$[CuAz]/[ZnAzd_5]$ or $[Co(III)]/[ZnAzd_5]$	Φ_{fluo}	Φ_{phos}	Φ_{rad}	Φ_{ET}
None	--	0.20 ± 0.01	0.013 ± 0.001	--	--
CuAzW48	2.2 ± 0.1	0.19 ± 0.01	0.0024 ± 0.0002	0.25 ± 0.02	0.27 ± 0.01
$[Co(NH_3)_5Cl]^{2+}$	2.2 ± 0.1	0.18 ± 0.01	<0.001	0.21 ± 0.01	--

Acceptor	$\Phi_{fluo}^{D}/\Phi_{fluo}^{H}$	$\Phi_{phos}^{D}/\Phi_{phos}^{H}$	$\Phi_{rad}^{D}/\Phi_{rad}^{H}$	$\Phi_{ET}^{D}/\Phi_{ET}^{H}$
None	0.95	0.72	--	--
CuAzW48	1.0	0.80	0.83	0.82
$[Co(NH_3)_5Cl]^{2+}$	0.95	not measurable	0.84	--

Figure S7. Normalized emission spectra of deoxygenated ZnAzW48 (blue) and ZnAzW48-*d*5 (red) with 270 nm excitation. The upper table shows Φ_{fluo}, Φ_{phos}, Φ_{ET}, and Φ_{rad} for samples with 23 ± 1 μM ZnAzW48-*d*5 (n = 3 for each row). The data from this upper table were compared to the data of protiated ZnAzW48 from Table 1 ($[CuAz]/[ZnAz] = 2.4$ and $[Co(III)]/[ZnAz] = 2.0$) to determine the isotope effects for fluorescence, phosphorescence, radical formation, and ET. Sample preparation for ZnAzW48-*d*5 has been previously described in Rivera *et al*., *J. Phys. Chem B.*, **2019**, 6430-6443.

Table S1. Φ_{fluo} and Φ_{phos} values for ZnAzW48 with $[Co(NH_3)_5Cl]^{2+}$ and CuAzW48 acceptors for representative trials at 10. °C and 22 °C.

Acceptor	$[Co(III)]/[ZnAz]$ or $[CuAz]/[ZnAz]$	Φ_{fluo}	Φ_{phos}	$\Phi^{o}_{phos}/\Phi_{phos}$	°C
None	--	0.20	0.019	--	22
None	--	0.19	0.026	--	10.
$[Co(NH_3)_5Cl]^{2+}$	0.26	0.19	0.010	1.8	22
$[Co(NH_3)_5Cl]^{2+}$	0.26	0.19	0.0036	7.2	10.
CuAzW48	0.51	0.19	0.0093	1.9	22
CuAzW48	0.57	0.20	0.013	2.0	10.

Table S2: Φ_{rad} and Φ_{ET} values for ZnAzW48 in the presence of both CuAzW48 and $[Co(NH_3)_5Cl]^{2+}$ acceptors.

Curve in Fig. 6	$[CuAz]/[ZnAz]$	$[Co(III)]/[ZnAz]$	Φ_{rad}	Φ_{ET}
Black square	1.3	0	0.25	0.26
Green circle	1.2	0.17	0.27	0.20
Red triangle	1.2	0.27	0.28	0.14
Blue star	1.3	1.2	0.25	0.035

Table S3: Quantum yield values for ZnAzW48 and apoAzW48.

Sample	Φ_{fluo}	Φ_{phos}
ZnAzW48	0.21 ± 0.02	0.018 ± 0.001
apoAzW48	0.21 ± 0.01	0.015 ± 0.001

Table S4: Quantum yield values for ZnAzW48 with $Cu(II)Cl_2$ quencher.

$[Cu(II)Cl_2]/[ZnAz]$	Φ_{fluo}	Φ_{phos}	Φ_{rad}
2.1	0.20	0.0013	0.14
28	0.19	<0.001	0.10

References:

(1) Muiño, P. L.; Callis, P. R. Solvent effects on the fluorescence quenching of tryptophan by amides via electron transfer. Experimental and computational studies. *J. Phys. Chem. B.* **2009,** *113* (9), 2572-2577.

(2) Parker, C. A.; Rees, W. T. Correction of fluorescence spectra and measurement of fluorescence quantum efficiency. *Analyst.* **1960,** *85* (1013), 587-600.

(3) Goldberg, M.; Pecht, I. Kinetics and equilibriums of the electron transfer between azurin and the hexacyanoiron (II/III) couple. *Biochemistry.* **1976,** *15* (19), 4197-4208.

(4) Larson, B. C.; Pomponio, J. R.; Shafaat, H. S.; Kim, R. H.; Leigh, B. S.; Tauber, M. J.; Kim, J. E. Photogeneration and quenching of tryptophan radical in azurin. *J. Phys. Chem. B.* **2015,** *119* (29), 9438-9449.
